# Supplementary material for: Protective mucosal immunity against SARS-CoV-2 after heterologous systemic prime-mucosal boost immunization
Source: Nat Commun. 2021 Nov 26;12:6871. doi: 10.1038/s41467-021-27063-4 (PMC8626513; doi:10.1038/s41467-021-27063-4)
Supplement: Supplementary file 3 — Reporting Summary [file 41467_2021_27063_MOESM3_ESM.pdf]

nature portfolio

Corresponding author(s): Matthias Tenbusch; Dennis Lapuente

Last updated by author(s): Oct 20, 2021

Reporting Summary

Nature Portfolio wishes to improve the reproducibility of the work that we publish. This form provides structure for consistency and transparency in reporting. For further information on Nature Portfolio policies, see our Editorial Policies and the Editorial Policy Checklist.

Statistics

For all statistical analyses, confirm that the following items are present in the figure legend, table legend, main text, or Methods section.

n/a	Confirmed	
☐	☒	The exact sample size (*n*) for each experimental group/condition, given as a discrete number and unit of measurement
☐	☒	A statement on whether measurements were taken from distinct samples or whether the same sample was measured repeatedly
☐	☒	The statistical test(s) used AND whether they are one- or two-sided<br>*Only common tests should be described solely by name; describe more complex techniques in the Methods section.*
☐	☒	A description of all covariates tested
☐	☒	A description of any assumptions or corrections, such as tests of normality and adjustment for multiple comparisons
☐	☒	A full description of the statistical parameters including central tendency (e.g. means) or other basic estimates (e.g. regression coefficient) AND variation (e.g. standard deviation) or associated estimates of uncertainty (e.g. confidence intervals)
☐	☒	For null hypothesis testing, the test statistic (e.g. F, t, r) with confidence intervals, effect sizes, degrees of freedom and P value noted<br>*Give P values as exact values whenever suitable.*
☒	☐	For Bayesian analysis, information on the choice of priors and Markov chain Monte Carlo settings
☒	☐	For hierarchical and complex designs, identification of the appropriate level for tests and full reporting of outcomes
☒	☐	Estimates of effect sizes (e.g. Cohen's *d*, Pearson's *r*), indicating how they were calculated

Our web collection on statistics for biologists contains articles on many of the points above.

Software and code

Policy information about availability of computer code

Data collection	BD FACS DIVA v6.1.2<br>ThermoFisher AttuneNxt v3.2.1526.0<br>PerkinElmer 2030 Manager software v4.00.015<br>CTL Immunospot v5.2.12
Data analysis	FlowJo v.10<br>GraphPad Prism v8.0

For manuscripts utilizing custom algorithms or software that are central to the research but not yet described in published literature, software must be made available to editors and reviewers. We strongly encourage code deposition in a community repository (e.g. GitHub). See the Nature Portfolio guidelines for submitting code & software for further information.

Data

Policy information about availability of data

All manuscripts must include a data availability statement. This statement should provide the following information, where applicable:

- Accession codes, unique identifiers, or web links for publicly available datasets
- A description of any restrictions on data availability
- For clinical datasets or third party data, please ensure that the statement adheres to our policy

All data available with the manuscript and a source data excel file is supplied with the manuscript.
NCBI Reference Sequence: NC_045512.2; https://www.ncbi.nlm.nih.gov/nuccore/1798174254
GISAID EPI ISL 406862 Germany/BavPat1/2020

Field-specific reporting

Please select the one below that is the best fit for your research. If you are not sure, read the appropriate sections before making your selection.

- [x] Life sciences
- [ ] Behavioural & social sciences
- [ ] Ecological, evolutionary & environmental sciences

For a reference copy of the document with all sections, see nature.com/documents/nr-reporting-summary-flat.pdf

Life sciences study design

All studies must disclose on these points even when the disclosure is negative.

Sample size	Sample size was determined by power analysis based on existing data sets from previous experiments with similar readouts and expected statistical errors (type I and II errors 0.05). Where no appropriate data sets existed, the minimal number of animals was choosen in order to produce data with sufficient statistical power.
Data exclusions	No data was excluded.
Replication	4-6 mice were included for each vaccination or control group. Immune responses, viral loads, and monitoring parameters upon infection were tested in all individual group animals. Some tests were repeated several times by different staff. All attempts at replication were successful. Immunization experiment 1 was not replicated because the data is consistent across the biological replicates and the main observations are statistically significant. Immunization experiment 2 was performed 3 times and the data was consistent across all experiments. T cell data cannot be pooled due to slighty different parameters of analyses. Antibody responses shown in Fig. 5 and 6 are from two independent immunizations and are consistent.
Randomization	Mice were randomly allocated to experimental groups by the animal care taker upon delivery. Randomization of samples is not relevant since analyses were done objectively by automatic devices (FACS, plate reader etc.).
Blinding	Blinding was not performed due to limited number of staff available to conduct these studies. Where possible, staff was blinded to groupings until after generation of the raw data.

Reporting for specific materials, systems and methods

We require information from authors about some types of materials, experimental systems and methods used in many studies. Here, indicate whether each material, system or method listed is relevant to your study. If you are not sure if a list item applies to your research, read the appropriate section before selecting a response.

Materials & experimental systems

n/a	Involved in the study	
	x	Antibodies
	x	Eukaryotic cell lines
x		Palaeontology and archaeology
	x	Animals and other organisms
x		Human research participants
x		Clinical data
x		Dual use research of concern

Methods

n/a	Involved in the study	
x		ChIP-seq
	x	Flow cytometry
x		MRI-based neuroimaging

Antibodies

Antibodies used	HRP-coupled anti-mouse IgA, Bethyl Laboratories, Cat: A90-103P, Lot: A90-103P-26

anti-mouse Ig-FITC; BD Biosciences, Cat: 554001, Lot: 8243834
anti-mouse IgG1-APC, clone X56, Biolegend, Cat:406610, Lot: B302145
anti-mouse IgG2a-FITC, clone R19-15, BD Biosciences, Cat: 553390, Lot: 8085987
anti-human IgG FITC 109-096-003, Jackson Immunoresearch
anti-CD45-BV510, clone 30-F11, Biolegend, Cat: 103138, Lot: B322199
anti-CD28, eBioscience, Cat: 14-0281-86, Lot:2023703
anti-CD107a-FITC, clone eBio1D4B, BD Bioscience, Cat: 553793, Lot: 0030739
anti-CD8a-Pacific blue, clone 53-6.7, BioLegend, Cat: 100725, Lot: B334776
anti-CD4-PerCP-eFluor710, clone RM4-5, eBioscience, Cat: 46-0042-82, Lot: 4317308
anti-IL-2-APC, clone JES6-5H4, BioLegend, Cat: 503810, Lot: B281632
anti-TNFα-PECy7, clone MPG-XT22, BioLegend, Cat: 506324, Lot: B309982
anti-IFNγ-PE, clone XMG1.2, BioLegend, Cat: 505808, Lot: B295804
anti-CD8-BV711, clone 53-6.7, BioLegend, Cat: 100784, Lot: B283373
anti-CD4-BV605, clone RM4-5, BioLegend, Cat: 100548, Lot: B284681
anti-CD127-FITC, clone A7R34, BioLegend, Cat: 135008, Lot: B255263
anti-CD69-PerCP-Cy5.5, clone H1.2F3, BioLegend, Cat: 104522, Lot: B287423
anti-CD103-PE, clone 2E7, eBioscience, Cat: 12-1031-82, Lot:4303132
anti-KLRG1-PE-Cy7, clone 2F1, eBioscience, Cat: 25-5893-82, Lot: 1982690
anti-CD44-APCAF647, clone IM7, BioLegend, Cat: 103018, Lot: B317762
anti-SARS-CoV-2 spike glycoprotein S1, clone CR3022, Cat: ab273073

Validation

All antibodies are commercially available and were validated by the manufacturer as follows:

HRP-coupled anti-mouse IgA, Bethyl Laboratories, Cat: A90-103P, Lot: A90-103P-26
The manufacturer provides the following statement: By immunoelectrophoresis and ELISA this antibody reacts specifically with mouse IgA. Cross reactivity to mouse IgM, IgG1, IgG2a, IgG2b, IgG2c, IgG3 and IgE is undetectable. The manufacturer states that the antibody was used in 41 publications.

anti-mouse Ig-FITC; BD Biosciences, Cat: 554001, Lot: 8243834
The manufacturer provides the following statement: Product routinely tested in flow cytometry.

anti-mouse IgG1-APC, clone X56, Biolegend, Cat:406610, Lot: B302145
The manufacturer provides the following statement: Each lot of this antibody is quality control tested by immunofluorescent staining with flow cytometric analysis. The manufacturer states that the antibody was used in 14 publications.

anti-mouse IgG2a-FITC, clone R19-15, BD Biosciences, Cat: 553390, Lot: 8085987
The manufacturer provides the following statement: Product routinely tested in flow cytometry. The manufacturer states that the antibody was used in 2 publications.

anti-human IgG FITC 109-096-003, Jackson Immunoresearch
The manufacturer provides the following statement: Based on immunoelectrophoresis and/or ELISA, the antibody reacts with whole molecule human IgG. It also reacts with the light chains of other human immunoglobulins.

anti-CD45-BV510, clone 30-F11, Biolegend, Cat: 103138, Lot: B322199
The manufacturer provides the following statement: Each lot of this antibody is quality control tested by immunofluorescent staining with flow cytometric analysis. The manufacturer states that the antibody was used in 66 publications.

anti-CD28, eBioscience, Cat: 14-0281-86, Lot:2023703
The manufacturer provides the following statement: Applications Tested: The 37.51 antibody has been tested by flow cytometric analysis of mouse splenocytes. The manufacturer states that the antibody was used in 59 publications.

anti-CD107a-FITC, clone eBio1D4B, BD Bioscience, Cat: 553793, Lot: 0030739
The manufacturer provides the following statement: Applications Tested: This antibody conjugate has been tested by intracellular immunofluorescent staining (≤ 1 µg/million cells, using the Cytofix/Cytoperm™ Kit, Cat. no. 554714) with flow cytometric analysis to assure specificity and reactivity. The manufacturer states that the antibody was used in 3 publications.

anti-CD8a-Pacific blue, clone 53-6.7, BioLegend, Cat: 100725, Lot: B334776
The manufacturer provides the following statement: Each lot of this antibody is quality control tested by immunofluorescent staining with flow cytometric analysis. The manufacturer states that the antibody was used in 38 publications.

anti-CD4-PerCP-eFluor710, clone RM4-5, eBioscience, Cat: 46-0042-82, Lot: 4317308
The manufacturer provides the following statement: This RM4-5 antibody has been tested by flow cytometric analysis of mouse spleen cells. This can be used at less than or equal to 0.125 µg per test. A test is defined as the amount (µg) of antibody that will stain a cell sample in a final volume of 100 µL. The manufacturer states that the antibody was used in 53 publications.

anti-IL-2-APC, clone JES6-5H4, BioLegend, Cat: 503810, Lot: B281632
The manufacturer provides the following statement: Each lot of this antibody is quality control tested by immunofluorescent staining with flow cytometric analysis. The manufacturer states that the antibody was used in 13 publications.

anti-TNFα-PECy7, clone MPG-XT22, BioLegend, Cat: 506324, Lot: B309982

The manufacturer provides the following statement: Each lot of this antibody is quality control tested by immunofluorescent staining with flow cytometric analysis. The manufacturer states that the antibody was used in 24 publications.

anti-IFNy-PE, clone XMG1.2, BioLegend, Cat: 505808, Lot: B295804
The manufacturer provides the following statement: Each lot of this antibody is quality control tested by immunofluorescent staining with flow cytometric analysis. The manufacturer states that the antibody was used in 98 publications.

anti-CD8-BV711, clone 53-6.7, BioLegend, Cat: 100784, Lot: B283373
The manufacturer provides the following statement: Each lot of this antibody is quality control tested by immunofluorescent staining with flow cytometric analysis. The manufacturer states that the antibody was used in 41 publications.

anti-CD4-BV605, clone RM4-5, BioLegend, Cat: 100548, Lot: B284681
The manufacturer provides the following statement: Each lot of this antibody is quality control tested by immunofluorescent staining with flow cytometric analysis. The manufacturer states that the antibody was used in 68 publications.

anti-CD127-FITC, clone A7R34, BioLegend, Cat: 135008, Lot: B255263
The manufacturer provides the following statement: Each lot of this antibody is quality control tested by immunofluorescent staining with flow cytometric analysis. The manufacturer states that the antibody was used in 8 publications.

anti-CD69-PerCP-Cy5.5, clone H1.2F3, BioLegend, Cat: 104522, Lot: B287423
The manufacturer provides the following statement: Each lot of this antibody is quality control tested by immunofluorescent staining with flow cytometric analysis. The manufacturer states that the antibody was used in 21 publications.

anti-CD103-PE, clone 2E7, eBioscience, Cat: 12-1031-82, Lot:4303132
The manufacturer provides the following statement: The 2E7 antibody has been tested by flow cytometric analysis of mouse splenocytes. This can be used at less than or equal to 1 µg per test. A test is defined as the amount (µg) of antibody that will stain a cell sample in a final volume of 100 µL. The manufacturer states that the antibody was used in 45 publications.

anti-KLRG1-PE-Cy7, clone 2F1, eBioscience, Cat: 25-5893-82, Lot: 1982690
The manufacturer provides the following statement: This 2F1 antibody has been tested by flow cytometric analysis of mouse splenocytes. This can be used at less than or equal to 0.25 µg per test. A test is defined as the amount (µg) of antibody that will stain a cell sample in a final volume of 100 µL. The manufacturer states that the antibody was used in 10 publications.

anti-CD44-APCAF647, clone IM7, BioLegend, Cat: 103018, Lot: B317762
The manufacturer provides the following statement: Each lot of this antibody is quality control tested by immunofluorescent staining with flow cytometric analysis. The manufacturer states that the antibody was used in 12 publications.

anti-SARS-CoV-2 spike glycoprotein S1, clone CR3022, Cat: ab273073
The manufacturer states that the antibody was used in 6 publications.

All antibodies were titrated in house to optimize specific staining.

Eukaryotic cell lines

Policy information about cell lines

Cell line source(s)	- HEK293T cells (ACC635, purchased from DSMZ; German Collection of Microorganisms and Cell Cultures GmbH)<br>- HEK293T-ACE2 generated in house by stable transduction of HEK293T cells leading to human ACE2 expression<br>- Vero E6 cells (C1008, Vero 76, clone E6, Vero E6; purchased from Sigma)
Authentication	None of the cell lines were authenticated since their purchase.
Mycoplasma contamination	Cell lines were all tested negative for mycoplasma contamination.
Commonly misidentified lines (See ICLAC register)	No commonly misidentified lines were used in this study.

Animals and other organisms

Policy information about studies involving animals; ARRIVE guidelines recommended for reporting animal research

Laboratory animals	- Mus musculus, BALB/cJRj, female, 6-8 weeks<br>- Mus musculus, C57BL/6, female, 6-8 weeks<br>- Mus musculus, K18-hACE2, female, 11 weeks
Wild animals	The study did not involve wild animals.
Field-collected samples	The study did not involve samples collected from the field.

Ethics oversight	In Germany, authorization of animal experiments is duty of the federal state administration (here the Government of Lower Franconia) who nominates an external ethics committee that advice and authorize animal studies for the government. The permission itself is granted by the local authority after positive vote from the ethics committee. The specific license numbers of the present study is given in the ethics statement at the beginning of the methods section.

Note that full information on the approval of the study protocol must also be provided in the manuscript.

Flow Cytometry

Plots

Confirm that:

- [x] The axis labels state the marker and fluorochrome used (e.g. CD4-FITC).
- [x] The axis scales are clearly visible. Include numbers along axes only for bottom left plot of group (a 'group' is an analysis of identical markers).
- [x] All plots are contour plots with outliers or pseudocolor plots.
- [x] A numerical value for number of cells or percentage (with statistics) is provided.

Methodology

Sample preparation	Spleens and lungs were harvested. The latter ones were cut into small pieces followed by incubation for 45 min at 37 °C with 500 units Collagenase D and 160 units DNase I in 2 ml R10 medium (RPMI 1640 supplemented with 10 % FCS, 2 mM L-Glutamine, 10 mM HEPES, 50 µM β-mercaptoethanol and 1 % penicillin/streptomycin). Digested lung tissues and spleens were mashed through a 70 µm cell strainer before the single cell suspensions were subjected to an ammonium-chloridepotassium lysis. One million splenocytes or 20% of the total lung cell suspension were plated per well in a 96-well round-bottom plate for in vitro restimulation and phenotype assays.
Instrument	- ThermoFisher AttuneNxt 4 Laser (violet, blue, yellow, red), 2018, Serial 2AFC219120118<br>- BD LSR II, 3 Laser (blue, violet, red), 2008, Model IC35480, Serial H47600111
Software	FlowJo v10<br>ThermoFisher AttuneNxt v3.1.2
Cell population abundance	No sorts in the manuscript
Gating strategy	Gating of antigen-experienced memory T cell subsets: Doublets were excluded by plotting FSC-H against FSC-A followed by gating on cells with roughly similar height and area. Next, lymphocytes were gated by plotting FSC-A against SSC-A. Small FSC debris was excluded as well as high SSC and high FSC events. Gating was checked by backgating of T lymphocytes. Dead lymphocytes were then excluded by staining for the used live-dead marker. Definition of stained/unstained cells was done with the help of unstained controls. Next, CD8 T cells were gated by plotting the CD4 against CD8 signal. The population was discrete and well defined. Unstained controls did not show any signal. Subsequently, CD44 positive cells were defined. Only cells highly positive for CD44 were selected, while dim and negative cells were not included. CD44+ CD8+ T cells were then divided into iv+ and iv- cells based on staining for the in vivo injected anti-CD45 antibody. Both populations were well separated from each other. TRM phenotypes were determined within the iv- population and the circulating phenotypes (TEFF, TEM, TCM) were defined within the iv+ population. TEFF and TEM were identified by their expression of KLRG1 and presence (TEM) or absence (TEFF) of CD127. KLRG1-negative cells were than gated and CD103 was plotted against CD69. TRM were defined as CD103+CD69+ as the most stringent identification of TRM cells. Lastly, CD69-CD103- cells were gated and TCM cells within this population were defined by their expression of CD127. CD127+ TCM were defined at an identical cut-off as for the TEM cells and this cut-off was determined in comparison to unstained controls.<br>Gating for intracellular cytokine staining after peptide-restimulation: Gating of singlets, lymphocytes, vital cells, CD4/CD8 T cells, and iv-/iv+ cells was similar as described above. Cytokine-producing cells were gated in parallel on the same hierarchy level for each analyzed parameter (CD107a, TNFa, IFNg, IL-2 for CD8; TNFa, IFNg, IL-2 for CD4), both in iv- and iv+ lung cells or in splenic T cells without consideration of the iv-staining. Control samples without stimulation, anti-CD3-stimulated samples, and controls without cytokine staining were used to set the gate for the respective cytokine-positive population. In order to quantify polyfunctional cells positive for all assessed markers, the implemented Boolean gating function of FlowJo was used.

- [x] Tick this box to confirm that a figure exemplifying the gating strategy is provided in the Supplementary Information.
